# Exploring the Potential of Enhanced Prognostic Performance of NCCN‐IPI in Diffuse Large B‐Cell Lymphoma by Integrating Tumor Microenvironment Markers: Stromal FOXC1 and Tumor pERK1/2 Expression

**DOI:** 10.1002/cam4.70305

**Published:** 2024-10-15

**Authors:** Ji‐Ye Kim, Ibadullah Kahttana, Hyonok Yoon, Sunhee Chang, Sun Och Yoon

**Affiliations:** ^1^ Department of Pathology, Ilsan Paik Hospital Inje University College of Medicine Goyang‐si Gyeonggi‐do Republic of Korea; ^2^ Department of Pathology Yonsei University College of Medicine, Severance Hospital Seoul Republic of Korea; ^3^ Division of Electronics and Information Engineering Jeonbuk National University Jeonju‐si Republic of Korea; ^4^ College of Pharmacy Research Institute of Pharmaceutical Sciences, Gyeongsang National University Jinju‐si Republic of Korea

**Keywords:** Diffuse Large B‐cell Lymphoma, FOXC1, Machine Learning Models, pERK1‐2, Prognostic Markers, Tumor Microenvironment

## Abstract

**Background:**

FOXC1 and ERK1‐2 are proteins implicated in aggressive biological behavior of various malignancies including lymphomas.

**Material and Methods:**

We investigate the additive prognostic value of stromal FOXC1 expression and tumor phosphorylated ERK1‐2 (pERK1‐2) expression to the established National Comprehensive Cancer Network International Prognostic Index (NCCN‐IPI), in 92 diffuse large B‐cell lymphoma (DLBCL) cases. Multidimensional analysis using statistics and machine learning (ML) models assessed prognostic value of established clinicopathologic variables with stromal FOXC1 and tumor pERK1‐2 expressions.

**Results:**

Both high FOXC1 stroma group and high pERK1‐2 tumor group were significantly associated with shorter progression‐free survival (PFS) and overall survival (OS) compared with low group (*p* = 0.015, 0.034 and *p* = 0.025, 0.025 each respectively). In multivariable analysis, high FOXC1 stromal expression was an independent prognostic factor of OS (*p* = 0.037). The addition of stromal FOXC1 and tumor pERK1‐2 to the NCCN‐IPI score significantly improved prediction of time to death compared with NCCN‐IPI score alone (Harrell's C‐index = 0.801 vs. 0.764; *p* = 0.030). ML models reconfirmed the addition of stromal FOXC1 expression and tumor pERK1‐2 to NCCN‐IPI score had the highest C‐index (0.952) among combinations. Stromal FOXC1 and tumor pERK1‐2 were determinants of DLBCL prognosis, whose addition significantly improved prognostic performance of the NCCN‐IPI.

## 1 Introduction

Diffuse large B‐cell lymphoma (DLBCL) is the most common non‐Hodgkin lymphoma, accounting for nearly 40%, with a heterogeneous clinical course [1]. The International Prognostic Index (IPI) and its more recent iteration, the National Comprehensive Cancer Network (NCCN)‐IPI, have served as established prognostic predictors for DLBCL for over three decades. However, both indices exhibit limitations in accurately forecasting the progression of DLBCL [2, 3, 4, 5, 6, 7]. Until recently, while the tumor alone has been the main focus in the study of oncology, there is increasing evidence that tumor‐stromal cell interactions play a critical role in the tumorigenesis and prognosis of DLBCL patients [7, 8, 9, 10]. A recent large‐scale study by Ruppert et al., analyzed survival outcomes of 2124 DLBCL patients and concluded that the existing clinical models to predict the clinical course of DLBCLs works with only moderate prognostic power [7]; they acknowledged that although National Comprehensive Cancer Network International Prognostic Index (NCCN‐IPI) performs best among existing prediction models with a concordance index (C‐index) of 0.632, integration of molecular features of the tumor microenvironment may improve prediction of survival.

In the context of the tumor microenvironment, cancer‐associated reticular cells (CARCs) have emerged as key players, particularly through their role in immune regulation [11, 12, 13]. CARCs, derived from fibroblastic reticular cells, form a network within secondary lymphoid organs that supports immune cell function. In lymphomas, these cells not only contribute to immune suppression by influencing the infiltration and activity of immune cells, but they also serve as cancer‐associated fibroblasts [12, 13]. These fibroblasts are responsible for matrix remodeling and the secretion of soluble factors that modulate cancer invasion, immune cell‐endothelial cell interactions, and tumor growth, thereby promoting a favorable environment for tumor progression. Their interaction with immune cells and secretion of cytokines and chemokines underscores their role in shaping the immune landscape within the tumor, potentially impacting patient prognosis and response to therapy.

FOXC1 is one of the forkhead family of transcription factors, characterized by a winged‐helix DNA‐binding forkhead domain [14]. FOX family proteins play a role in many biological processes within the cell, including proliferation, differentiation, survival, and death. FOXC1 expression has been studied in epithelial tumor cells of numerous malignancies, such as the breast, bladder, lung, uterus, liver, and the stomach, promoting fibrosis and epithelial‐to‐mesenchymal transition by regulating FGFR thereby influencing tumor development and progression [14, 15, 16, 17]. However, in the field of hematologic malignancies, very few studies have been conducted, which may be due to the lack of expression of FOXC1 on lymphoid cells [17, 18, 19, 20]. Omatsu et al. discovered that FOXC1 was specifically expressed within reticular cells of mouse bone marrow, which maintain hematopoietic stem/progenitor cells [19]. Consistent with such findings, our previous work on T and NK cell lymphomas revealed that FOXC1 was preferentially expressed in stromal cells of the lymphoma environment [18]. One rare study on DLBCLs found that the bulk RNA expression of FOXC1 correlated with secondary extranodal localization, indicating poor prognosis [20]. Taken together, we hypothesize that FOXC1 expression, primarily originating from stromal cells within the DLBCL tumor microenvironment, contributes to the aggressive biological behavior of DLBCLs.

Extracellular signal‐regulated kinase 1 and 2 (ERK1‐2) belongs to the mitogen‐activated protein kinases (MAPK) family of highly conserved intracellular kinases, in which the active form is phosphorylated ERK1‐2 (pERK1‐2). They are best known for transducing extracellular signals relayed by surface receptors or various types of damage, playing crucial roles in various cellular processes, including proliferation, differentiation, and survival. The role of the MAPK/ERK pathway in cancer is well‐documented across a wide range of malignancies including DLBCL [21, 22, 23, 24, 25, 26]. Moreover, targeting ERK1‐2 for DLBCL treatment has been a subject of constant exploration [27, 28, 29]. A study by Nguyen et al. demonstrated that inhibition of ERK1‐2 by the multikinase inhibitor, Sorafenib increased apoptosis in DLBCL cells regardless of the molecular subtype by inhibiting the antiapoptotic protein MCL‐1, thereby activating the apoptotic pathway [28]. These evidences support that pERK1‐2 expression in DLBCL strongly correlates with proliferation and extended survival leading to aggressive behavior. Moreover, Berry et al. had discovered that the ERK1/2 MAPK signaling pathway is required for maximal FOXC1 transcriptional activation and stability within HeLa cells [30]. However, FOXC1 in stromal cells and pERK1‐2 in tumoral DLBCL cells in the context of DLBCL prognosis has not been explored.

Incorporation of machine learning (ML) models into survival analysis is a relatively new, yet rapidly expanding approach in research. Its application has become prevalent in the field of oncology, facilitating study of various cancer survival models, including breast, lung, esophageal, and brain cancers [31, 32, 33]. ML models have demonstrated their ability to complement and sometimes surpass the traditional Cox proportional hazards model, which has been a cornerstone in this field [31, 32, 33]. While classical statistics excel in elucidating relationships between variables, ML offers greater adaptability, performance, and capacity for managing complex data structures. A notable limitation of the Cox model is its assumption that the effect of covariates on hazard rate remains constant over time [34], an assumption often untenable in biological contexts. In contrast, ML models are not constrained by the proportional hazards assumption, enabling them to more effectively model time‐varying effects. Recognizing the strengths of both analytical techniques, we employ both classical statistics and ML models for a comprehensive analysis of our hypothesis.

We hypothesized that FOXC1 and pERK1‐2 would be prognostically significant in the setting of DLBCLs and their addition to established prognostic indices, that is, NCCN‐IPI, would improve prognostic performance. With these novel molecules, we explore their possible roles in the setting of DLBCL which may further understanding of aggressively behaved DLBCLs and provide foundations for targeted treatment and better prognostic guidance. Employing traditional statistical methods and ML techniques, we provide an in‐depth analysis of the prognostic value of FOXC1 expression in stromal cells and pERK1‐2 expression in DLBCL tumor cells with established clinicopathological factors.

## 2 Methods

### 2.1 Patient and Tissue Samples

Archived formalin‐fixed, paraffin‐embedded tissue samples were obtained from 134 patients who had DLBCL, diagnosed from 2005 to 2013 at Severance Hospital. Among cases, 93.5% had cyclophosphamide, doxorubicin, vincristine, prednisone, and rituximab (R‐CHOP) therapy or R‐CHOP‐like therapy. All samples were collected from the primary site where 34 (37.4%) cases were from lymph nodes and 58 (62.6%) cases from extranodal sites. Ten normal tonsil tissues from cancer‐free individuals were used for normal controls. The histopathology of all cases was reviewed by two pathologists (K. J. and Y. S. O.) according to 2008 World Health Organization (WHO) criteria [35]. The Institutional Review Board (IRB) of Yonsei University Severance Hospital approved the study (2019‐1925‐001).

### 2.2 Tissue Microarray Preparation and Immunohistochemistry

The representative tumor areas in each case were selected by microscopic examination to build tissue microarrays (TMAs). Two or three different representative areas per case were selected and core tissues (3 mm in diameter) were taken from the individual formalin‐fixed paraffin‐embedded (FFPE) blocks (donor blocks) and arranged in recipient paraffin blocks (tissue array blocks) using a trephine apparatus. All TMA blocks were confirmed to contain suitable tumor lesion, each occupying more than 50% of core area under the microscope.

Immunohistochemistry was performed on 4 μm TMA tissue sections using Ventana Bench Mark XT Autostainer (Ventana Medical Systems, Tucson, AZ, USA) and LEICA BOND‐III Autostainer and Bond Polymer Refine Detection kit (Leica Biosystems). The tested primary antibodies were followings: FOXC1 (dilution 1:50, ab5079; Abcam, Cambridge, UK), pERK1‐2 (dilution 1:100, clone #4370; Cell Signaling Technology, Beverly, MA), c‐MYC (dilution 1:50, clone Y69; Abcam, Cambridge, UK), the Novolink kit (Leica Biosystems, Newcastle Upon Tyne, UK), BCL2 (dilution 1:50; Novocastra, Newcastle, UK), CD10 (dilution 1:100; Novocastra), BCL6 (RTU; Novocastra), MUM1 (dilution 1:200; Cell Marque, Rocklin, CA), and Ki‐67 (clone MIB‐1, DAKO, Glostrup, Denmark). For in situ hybridization for Epstein–Barr virus (EBV), the INFORM EBER probe (Ventana Medical Systems, Tucson, AZ) was used with the Ventana BenchMark XT Autostainer (Ventana Medical Systems) and ISH iVIEW Blue Detection Kit (Ventana Medical Systems).

### 2.3 Counting, Scoring, and Interpretation of Markers

Hematoxylin and eosin‐stained slides were examined microscopically to localize DLBCL tumor cells and stromal cells within the tissue field. The stromal cells were morphologically identified by star‐shaped dendritic cells with relatively abundant cytoplasm, distinct from DLBCL cells. Each field was packed with tumor and tumor microenvironment cells containing 800–1000 cells per ×400 magnification high power field (HPF) with minimal intervening matrix. FOXC1 had the preference of choice for the selection of fields; FOXC1 was first examined to select four fields that had the most staining FOXC1 stromal cells for each case. Then, the same exact fields were selected for pERK. Each field of examination was photographed to assure consistency of the field of examination at a given time. FOXC1 preferentially stained stromal cells rather than tumor cells. Stromal cells that had moderate‐to‐strong staining were counted as positive; stromal cells with no staining to weak staining were excluded. FOXC1 staining stromal cells were counted manually using the counter application of cellSens image acquisition program (Olympus scientific solutions). pERK1‐2 expression was counted positive in those with nuclear staining. pERK1‐2 staining was observed in both stromal and tumor cells; however only positive staining tumor cells were considered. Tumor cells that had moderate‐to‐strong staining were counted as positive; tumor cells with no staining to weak staining were excluded. pERK1‐2‐positive tumor cells were recorded as the percentage of the total tumor cells present within each field. From the mean value of four fields, an optimal cutoff point was determined by log‐rank analysis of overall survival. The cutoff for the high expression group for the markers were, > 18 FOXC1‐positive stromal cells/HPF and > 2.5% pERK‐positive tumor cells.

For CD10, BCL6, and MUM1, the positive cutoff value was determined according to the Hans classification criteria [36] and was considered positive if ≥ 30% of the tumor cells showed nuclear immunoactivity for BCL6 and MUM1, and if ≥ 30% of cells showed membranous reactivity for CD10. Determination of the germinal center B‐like (GCB) or non‐GCB phenotype was based on the Hans algorithm [36]. For c‐MYC and BCL2, the threshold value was determined for c‐MYC when ≥ 40% of tumor cells revealed moderate‐to‐strong expression, and BCL2 when ≥ 70% of tumor cells revealed moderate‐to‐strong expression [37, 38]. For Ki‐67, the percentage of tumor cells which showed positive expression was scored. For EBV in situ hybridization, the threshold value was determined when ≥ 10% of the tumor cells showed moderate‐to‐strong nuclear expression.

### 2.4 Multivariate Overall Survival Modeling via Machine Learning

**FIGURE 1:**
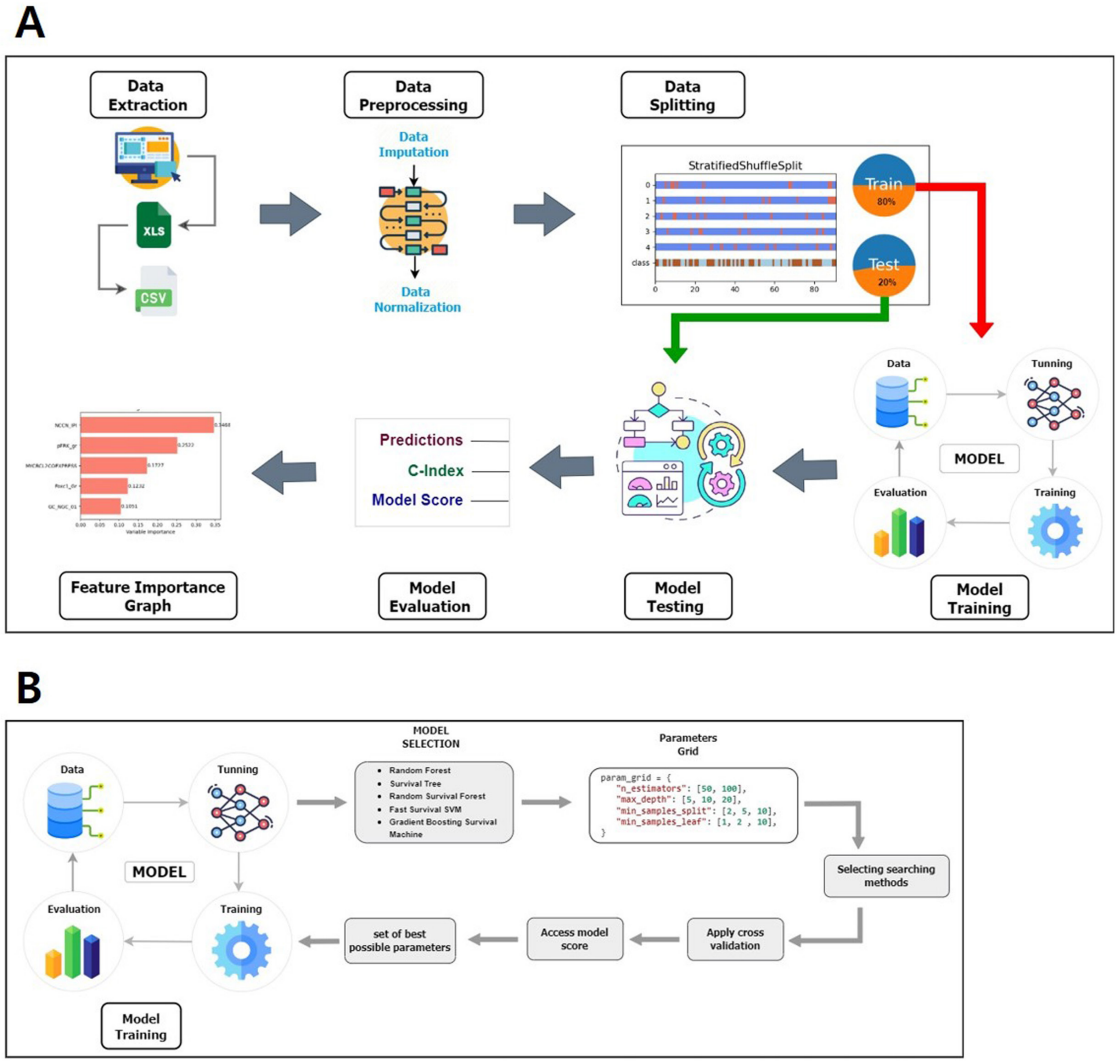
Machine learning modeling system workflow. (A) The overall workflow of the proposed modeling system. (B) The workflow of the hyperparameter tuning process.

Before modeling, the input datasets were in the form of categorical data. The output value, time to death, was kept as a continuous value. For the purpose of survival analysis, we employed various preprocessing rules to transform our time‐to‐event data and associated event information into a format compatible with ML methods. The modeling system workflow was conducted in seven stages as depicted in Figure 1A, which outlines the overall workflow of the modeling system architecture. First, the dataset was extracted from the original Excel file for conversion into a simple CSV file format. Next, the data were preprocessed by normalization using Min‐Max normalization technique to scale all datasets to a range between zero and one.

(1) $$X_{\mathrm{norm}}=\frac{X-X_{\min}}{X_{\max}-X_{\min}}$$

In Equation [1], X is our feature vector, $X_{\max}$ is the maximum feature vector, $X_{\min}$ is the minimum feature vector, and $X_{\mathrm{norm}}$ is the normalized value. The Min‐Max normalization technique provides a usual scale for all numerical features, to prevent bias toward variables with large scales. Next, the data splitting technique was employed to balance the splitting of data into training (80%) and testing (20%) sets. To achieve proportional class representation, the Stratified Shuffle Split technique with 5‐folds cross‐validation was used. This approach systematically divided the training portion into quintiles, sequentially designating a single fold as the validation set while utilizing the remainder for model fitting. We repeat the technique five times, with each iteration featuring a different fold as the validation subset. For training and testing, the following list of 6 ML algorithms specific to survival analysis were used.

1. ML methods: Fast Survival Support Vector Machine (FastSVM) [39], Random Survival Forest (RF‐Survival) [40], and Survival Tree [41].
2. ML Ensemble methods: Extreme Gradient Boosting (XGBoost) [42] with tree‐based boosting, Gradient Boosting Survival Analysis (GBoostSurv) [43], and Bagging Survival Trees with bootstrap aggregated survival function (Bagging SurvTree) [44].

To prioritize features based on a certain criterion, nine feature selection techniques that could handle high‐dimensional data were used: Univariate statistical feature selection test (SelectKBest) [45], Random Forest Variable of Importance (RF Var Imp) [46], Random Forest Minimum‐Depth Importance (RF Min Depth) [47], Random Forest Maximum Rank Statistics (RF Max Stat) [48], Minimum‐Redundancy and Maximum‐Relevance (mRMR) [49], Recursive Feature Elimination (rFE), and Sequential Forward Selection (SFS) [50]. Each feature selection method was employed for each survival model where possible.

Prior to the training phase, the hyperparameter tuning method, GridSearchCV, was applied to identify the optimal hyperparameters for each model in the study. Utilizing GridSearchCV involves specifying a list of hyperparameters and performance metrics. The algorithm then systematically explores all possible combinations of these parameters to determine the most suitable parameters that maximize performance. Given that we had six survival analysis models and 16 different combination sets of input features, we performed hyperparameter tuning for a total of 96 times. Figure 1B illustrates the workflow of the hyperparameter tuning process.

The C‐index was used to access each algorithm's prediction ability. Upon training and testing, the model's predictive outputs were harnessed.

### 2.5 Statistical Analysis

For the determination of statistical significance, two‐sample *t*‐test or one‐way analysis of variance (ANOVA) was used for continuous variables. When analyzing data with multiple comparisons, a corrected *p* value with application of Bonferroni multiple comparison procedure was used. Correlation between variables was analyzed through Spearman's rank correlation coefficient due to the skewed nature of all three variables. An optimal cutoff point was determined by receiver operator curves and Youden's index characteristic curves and Youden's index. Statistical significance was set to *p* < 0.05. Kaplan–Meier survival curves and log‐rank statistics were used to evaluate survival rate. Cox proportional hazards model was used for multivariate regression analysis and Harrell's C‐index. Data were analyzed using SPSS for Windows, Version 21.0 (SPSS Inc., Chicago, IL, USA).

## 3 Results

### 3.1 FOXC1 Stromal Cell and pERK1‐2 Tumor Cell Expression in the Tumor Microenvironment

**FIGURE 2:**
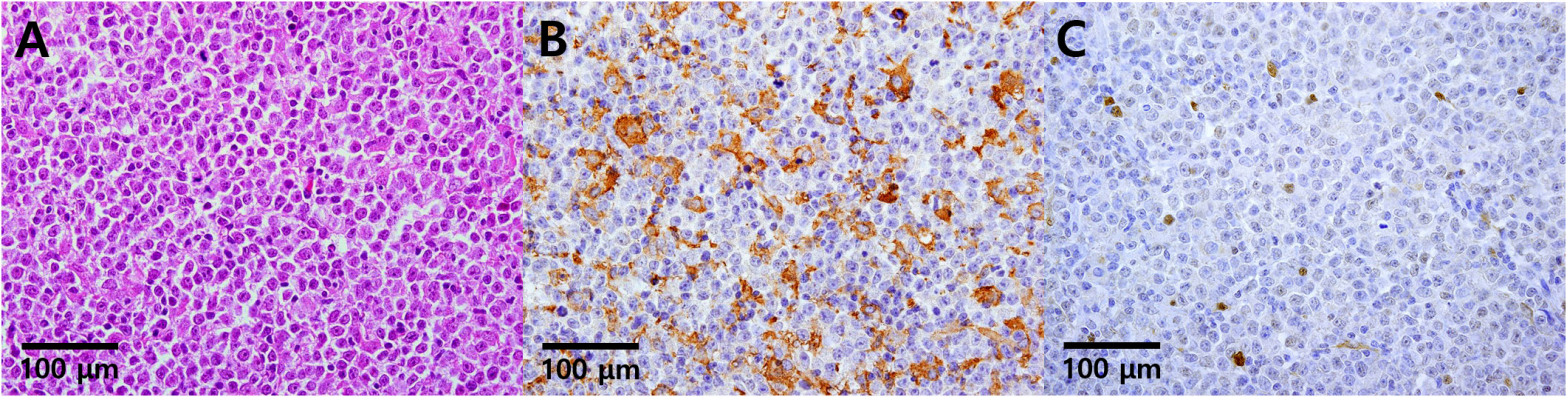
Distinctive staining patterns of stromal FOXC1 and tumoral pERK1‐2 expression in DLBCL patient tissue samples. (A) Representative photomicrograph of the hematoxylin–eosin section of a DLBCL patient on high power (×20, objective). The tumor microenvironment is composed of the predominant aggregate of large tumoral B cells and interspersed stromal cells. (B) Representative photomicrographs showing the preferential FOXC1 immunoreactivity for stromal cells. (C) pERK1‐2 immunoreactivity was observed in both stromal cells and tumor cells.

In four cases of control normal tonsillar tissue, FOXC1 was frequently expressed in the dendritic stromal cells (average 44.25 cells/HPF) while pERK1‐2 had rare expression on normal lymphocytes (average < 1%). On DLBCL patient tissue, FOXC1 expression was preferentially observed in the cytoplasm of star‐shaped stromal cells within the tumor microenvironment and rarely in tumor cells; pERK1‐2 expression was counted in the DLBCL tumor cell nucleus (Figure 2).

The number of FOXC1‐positive stromal cells and pERK1‐2‐positive tumor cells varied among cases. Maximum number of stromal FOXC1‐expression per HPFs was 59.750, minimum 0, and mean 16.523 cells. Maximum percentage of the pERK1‐2 expression per HPFs was 77%, minimum 0%, and mean 13.533%. No significant correlation was observed between FOXC1‐positive stromal cells and pERK1‐2‐positive tumor cells (*R* = 0.13, *p* = 0.208).

### 3.2 Correlation of FOXC1‐Positive Stromal Cells and pERK1‐2‐Positive Tumor Cells With Clinicopathological Characteristics

In association with known clinicopathologic parameters, the status of FOXC1‐positive stromal cells and pERK1‐2‐positive tumor cells within the DLBCL tumor microenvironment is summarized in Table 1.

**TABLE 1:** Clinicopathological significance of stromal FOXC1 and tumoral pERK1‐2 expression in DLBCL patients.

	Stromal FOXC1			Tumoral pERK1‐2		
	Low	High	*p*	Low	High	*p*
NCCN‐IPI score items						
Age (years)						
< 41	13 (23.2)	6 (17.1)	0.844	6 (16.7)	13 (23.6)	0.606
41–60	20 (35.7)	15 (42.9)		17 (47.2)	18 (32.7)	
61–75	20 (35.7)	13 (37.1)		12 (33.3)	21 (38.2)	
> 75	3 (5.4)	1 (2.9)		1 (2.8)	3 (5.5)	
Clinical stage (Ann‐Arbor)						
I–II	36 (64.3)	13 (37.1)	**0.021** *	22 (61.1)	27 (49.1)	0.363
III–IV	20 (35.7)	22 (62.9)		14 (38.9)	28 (50.9)	
LDH ratio						
< ULN	35 (62.5)	15 (42.9)	0.186	21 (58.3)	29 (52.7)	0.762
1‐3× ULN	17 (30.4)	16 (45.7)		13 (36.1)	20 (36.4)	
> 3× ULN	4 (7.1)	4 (11.4)		2 (5.6)	6 (10.9)	
Extranodal disease^a^						
None	42 (75.0)	24 (68.6)	0.669	25 (69.4)	41 (74.5)	0.770
Extranodal	14 (25.0)	11 (31.4)		11 (30.6)	14 (25.5)	
ECOG PS						
< 2	50 (89.3)	29 (82.9)	0.526	32 (88.9)	47 (85.5)	0.758
≥ 2	6 (10.7)	6 (17.1)		4 (11.1)	8 (14.5)	
NCCN‐IPI risk group						
Low (0–1)	19 (33.9)	6 (17.1)	0.068	14 (38.9)	11 (20.0)	0.188
Low‐intermediate (2–3)	21 (37.5)	18 (51.4)		12 (33.3)	27 (49.1)	
High‐intermediate (4–5)	15 (26.8)	7 (20.0)		9 (25.0)	13 (23.6)	
High (6–7)	1 (1.8)	4 (11.4)		1 (2.8)	4 (7.3)	
Cell of origin^b^						
GCB	18 (32.1)	8 (23.5)	0.526	16 (44.4)	10 (18.5)	**0.015** *
NGC	38 (67.9)	26 (76.5)		20 (55.6)	44 (81.5)	
BCL2						
Negative	35 (64.8)	21 (61.8)	0.951	17 (48.6)	39 (73.6)	**0.031** *
Positive	19 (35.2)	13 (38.2)		18 (51.4)	14 (26.4)	
C‐MYC						
Negative	38 (70.4)	23 (67.6)	0.974	27 (77.1)	34 (64.2)	0.290
Positive	16 (29.6)	11 (32.4)		8 (22.9)	19 (35.8)	
MYC/BCL2 double‐exp.						
Negative	46 (85.2)	30 (88.2)	0.760	30 (85.7)	46 (86.8)	1.000
Positive	8 (14.8)	4 (11.8)		5 (14.3)	7 (13.2)	

* Statistically significant *p* values are highlighted in bold.

^a^ Extranodal disease defined as, any extralymphatic involvement of bone marrow, CNS, liver, lung, and/or GI tract.

^b^ Cell of origin assessed using Hans' algorithm via immunohistochemical staining.

Abbreviations: ECOG PS, Eastern Cooperative Oncology Group Patient's performance Status; GCB, germinal center B‐cell like; LDH, lactate dehydrogenase; MYC/BCL2 double‐exp., MYC/BCL2 double expression; NCCN‐IPI, National Comprehensive Cancer Network International Prognostic Index; NGC, nongerminal center B‐cell like; ULN, upper limit of normal.

Higher levels of FOXC1‐positive stromal cells were significantly associated with advanced Ann‐Arbor stage (III‐IV) (*p* = 0.021). High FOXC1‐positive stromal cells were not associated with cell of origin (germinal center type or nongerminal center type) and MYC/BCL2 double expression. High expression of pERK1‐2‐positive tumor cells were significantly associated with the nongerminal center cell type and negative BCL2 expression (*p* = 0.015 and 0.031, each respectively).

### 3.3 FOXC1‐Positive Stromal Cell and pERK1‐2‐Positive Tumor Cell Expression Correlates With Patient Survival

**FIGURE 3:**
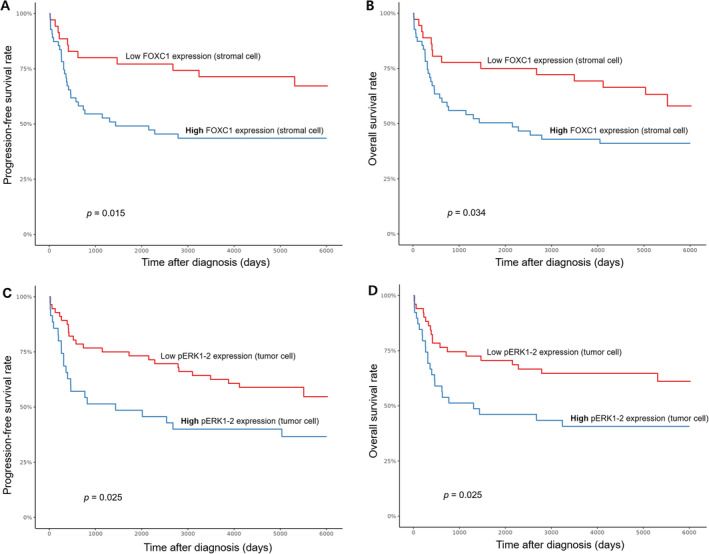
Kaplan–Meier plots 92 patients with DLBCL. (A) PFS and (B) OS of stromal FOXC1 expression groups. (C) PFS and (D) OS of tumoral pERK1‐2 expression groups.

The median follow‐up duration of DLBCL patients in our cohort was 12.3 (range: 15 days−19.9 years) years. The median PFS and OS were 46.5 (range, 7 days−8.2 years) months and 46.8 (range, 7 days−8.2 years) months each, respectively. FOXC1 stromal cell expression and pERK1‐2 tumor cell expression correlated with a significant decrease in both PFS and OS by log‐rank and multivariable analysis. Log‐rank test revealed that high FOXC1‐positive stromal cell group (> 18 FOXC1‐positive stromal cells/HPF) and high pERK1‐2‐positive tumor cell group (> 2.5% pERK1‐2 positive tumor cells) was significantly associated with shorter overall and progression‐free survival compared with low FOXC1‐positive stromal cell group (≤ 18 FOXC1‐positive stromal cells/HPF; *p* = 0.015 and *p* = 0.034, each respectively; Figure 3A,B) and low pERK1‐2‐positive tumor cell group (≤ 2.5% pERK1‐2‐positive tumor cells; both *p* = 0.025; Figure 3C,D). 39.1% (36/92) and 60.9% (56/92) of DLBCL cases were included in the high group for FOXC1 and pERK1‐2, each respectively. For FOXC1, the median PFS and OS were and 25.7 (range, 13.2–NA) months and 47.9 (range, 13.2–NA) months, respectively; for pERK1‐2, the median PFS and OS were and 47.9 (range, 19.3–NA) months and 76.1 (range, 24.6–NA) months, respectively. By multivariable analyses, high FOXC1‐positive stromal cell group was an independent prognostic factor of inferior overall survival (hazard ratio, 2.32; 95% CI, 1.25–4.33, *p* = 0.037, Table 2) along with low‐intermediate, high‐intermediate, and high NCCN‐IPI risk groups (hazard ratio, 5.13, 13.51, and 48.05; 95% CI, 1.49–17.71, 3.82–47.70, and 9.41–245.29; *p* = 0.010, < 0.001, and < 0.001, each respectively). Noteworthy is that although high FOXC1‐positive stromal cell group had statistical significance in multivariable overall survival, established clinical prognostic factors including, cell of origin and MYC/BCL2 double expression did not (both *p* = 0.189). Tumor pERK1‐2 expression had statistical significance only in univariable progression‐free and overall survival analysis (hazard ratio, 2.35 and 2.02; 95% CI, 1.18–4.70 and 1.08–3.80; *p* = 0.015 and 0.029; Table 2).

**TABLE 2:** Multivariable Cox proportional hazards model of stromal cell FOXC1 and tumor cell pERK1‐2 expression, NCCN‐IPI risk group, cell of origin, and MYC/BCL2 double expression in DLBCL patients (*n* = 92).

Variable	Progression‐free survival				Overall survival			
	Univariable		Multivariable		Univariable		Multivariable	
	HR (95% CI)	*p*	HR (95% CI)	*p*	HR (95% CI)	*p*	HR (95% CI)	*p*
pERK 1–2 (tumor cell)								
Low	1		1		1		1	
High	2.35 (1.18–4.70)	0.015*	1.28 (0.60–2.71)	0.522	2.02 (1.08,3.80)	0.029*	1.30 (0.65,2.62)	0.463
FOXC1 (stromal cell)								
Low	1		1		1		1	
High	1.99 (1.08–3.64)	0.027*	1.95 (0.96–3.80)	0.065	1.94 (1.09,3.47)	0.025*	2.32 (1.25,4.33)	**0.037** *
NCCN‐IPI risk group								
Low (0–1)	1		1		1		1	
Low‐intermediate (2–3)	9.09 (2.12–39.01)	0.003*	7.02 (1.59–31.00)	**0.010** *	6.44 (1.92–21.65)	0.003*	5.13 (1.49–17.71)	**0.010** *
High‐intermediate (4–5)	18.36 (4.16–81.03)	< 0.001*	15.95 (5.52–72.20)	**< 0.001** *	13.91 (4.02–48.1)	< 0.001*	13.51 (3.82–47.70)	**< 0.001** *
High (6–7)	77.25 (13.96–427.33)	< 0.001*	59.46 (9.68–365.35)	**< 0.001** *	68.31 (14.73–316.74)	< 0.001*	48.05 (9.41–245.29)	**< 0.001** *
Cell of origin^a^								
GCB	1				1			
NGC	2.11 (0.98–4.57)	0.057			1.57 (0.80–3.10)	0.189		
MYC/BCL2 double‐exp.								
Negative	1		1		1			
Positive	2.21 (1.05–4.65)	0.036*	1.45 (0.65–3.22)	0.610	1.98 (0.95–4.11)	0.189		
No. of observations	87				88			
No. of events	43				46			

* Statistically significant. Multivariable statistically significant *p* values are highlighted in bold.

^a^ Cell of origin assessed using Hans' algorithm via immunohistochemical staining.

Abbreviations: CI, confidence interval; GCB, germinal center B‐cell like; MYC/BCL2 double‐exp., MYC/BCL2 double expression; NCCN‐IPI, National Comprehensive Cancer Network International Prognostic Index; NGC, nongerminal center B‐cell like.

### 3.4 Addition of FOXC1‐Positive Stromal Cell and pERK1‐2‐Positive Tumor Cell Expression Improved Prognostic Performance of NCCN‐IPI by Multidimensional Analysis

To address whether the addition of stromal FOXC1 and tumor pERK1‐2 expression could improve prediction of recurrence or death of NCCN‐IPI, both classical statistics and ML models were employed.

By classical statistics, that is, Harrell's C‐index, the single addition of stromal FOXC1 or tumor pERK1‐2 to NCCN‐IPI improved the C‐index for the prediction of time to recurrence and death compared with NCCN‐IPI alone, although only tumor pERK1‐2 reached statistical significance in prediction of time to death (0.798 vs. 0.764, *p* = 0.038, Table 3). The addition of both stromal FOXC1 and tumor pERK1‐2 to NCCN‐IPI significantly improved C‐index than NCCN‐IPI alone in the prediction of time to death, (0.801 vs. 0.764, *p* = 0.030). Noteworthy is the addition of the established clinical prognostic factors of cell or origin and MYC/BCL2 double expression to NCCN‐IPI did not show significant difference in C‐index for both time to recurrence or death; these findings indicate that stromal FOXC1 and tumor pERK1‐2 expression have stronger association with the tumor biology than established prognostic factors.

**TABLE 3:** Harrell's C‐index for prediction of time to recurrence and time to death.

	Time to recurrence		Time to death	
Prediction model	C‐index (95% CI)	*p*	C‐index (95% CI)	*p*
NCCN‐IPI score	0.776 (0.714–0.839)	0.350	0.764 (0.705–0.823)	0.070
NCCN‐IPI score + stromal FOXC1	0.791 (0.729–0.853)		0.790 (0.732–0.849)	
NCCN‐IPI score	0.776 (0.714–0.839)	0.194	0.764 (0.705–0.823)	**0.038** *
NCCN‐IPI score + tumor pERK1‐2	0.801 (0.739–0.863)		0.798 (0.739–0.857)	
NCCN‐IPI score	0.776 (0.714–0.839)	0.121	0.764 (0.705–0.823)	**0.030** *
NCCN‐IPI score + stromal FOXC1 + tumor pERK1‐2	0.808 (0.745–0.870)		0.801 (0.742–0.860)	
NCCN‐IPI score	0.773 (0.710–0.837)	0.953	0.761 (0.699–0.823)	0.499
NCCN‐IPI score + cell of origin^a^ + MYC/BCL2 double‐exp.	0.774 (0.711–0.838)		0.769 (0.707–0.831)	
NCCN‐IPI score + cell of origin^a^ + MYC/BCL2 double‐exp.	0.774 (0.713–0.836)	0.345	0.769 (0.707–0.831)	0.113
NCCN‐IPI score + cell of origin^a^ + MYC/BCL2 double‐exp. + stromal FOXC1	0.791 (0.730–0.852)		0.793 (0.731–0.855)	
NCCN‐IPI score + cell of origin^a^ + MYC/BCL2 double‐exp.	0.774 (0.713–0.836)	0.151	0.769 (0.707–0.831)	**0.045** *
NCCN‐IPI score + cell of origin^a^ + MYC/BCL2 double‐exp. + tumor pERK1‐2	0.789 (0.728–0.850)		0.791 (0.729–0.853)	
NCCN‐IPI score + cell of origin^a^ + MYC/BCL2 double‐exp.	0.774 (0.713–0.836)	0.098	0.769 (0.707–0.831)	0.057
NCCN‐IPI score + cell of origin^a^ + MYC/BCL2 double‐exp. + stromal FOXC1 + tumor pERK1‐2	0.803 (0.742–0.864)		0.800 (0.738–0.862)	

* Statistically significant *p* values are highlighted in bold.

^a^ Cell of origin assessed using Hans' algorithm via immunohistochemical staining.

Abbreviations: C‐index, concordance index; CI, confidence interval; MYC/BCL2 double‐exp., MYC/BCL2 double expression; NCCN‐IPI, National Comprehensive Cancer Network International Prognostic Index.

Analysis by ML models and methods confirmed the findings that the addition of both stromal FOXC1 and tumor pERK1‐2 to established factors of prognosis (NCCN‐IPI, cell of origin, and MYC/BCL2 double expression) improved C‐index. We computed every combination of prognostic factors including established factors, stromal FOXC1 and tumor pERK1‐2‐to calculate the C‐index. A heat map was generated for each combination to overview performance (Figure S1). All indices of C‐index improved from the addition of both stromal FOXC1 and tumor pERK1‐2 to established factors of prognosis (Table 4); average C‐index of ML improved from 0.807 to 0.841 and the maximum C‐index from 0.921 to 0.939 (Table 4). The highest maximum C‐index among all combinations was 0.952 by NCCN‐IPI, cell of origin and tumor pERK‐1‐2 using Bagging SurvTree model and the RF min Depth method. Among the 16 combinations of inputs by six ML models, the Bagging SurvTree model constantly generated the highest C‐index.

**TABLE 4:** C‐index by machine learning models for input combinations.

Inputs	Avg. C‐index	Max. C‐index
NCCN‐IPI	0.769	0.867
NCCN‐IPI + stromal FOXC1	0.772	0.891
NCCN‐IPI + tumor pERK1‐2	0.829	0.878
NCCN‐IPI + cell of origin^a^ + MYC/BCL2 double‐exp.	0.807	0.921
NCCN‐IPI + stromal FOXC1 + tumor pERK1‐2	0.829	**0.952** *
NCCN‐IPI + cell of origin^a^ + MYC/BCL2 double‐exp. + stromal FOXC1	0.806	0.894
NCCN‐IPI + cell of origin^a^ + MYC/BCL2 double‐exp. + tumor pERK1‐2	**0.846** *	0.894
NCCN‐IPI + cell of origin^a^ + MYC/BCL2 double‐exp. + stromal FOXC1 + tumor pERK1‐2	0.841	0.939

* Maximum value of each index is highlighted in bold.

^a^ Cell of origin assessed using Hans' algorithm via immunohistochemical staining.

Abbreviations: Avg., average; C‐index, concordance index; CI, confidence interval; GCB, germinal center B‐cell like; Max., maximum; MYC/BCL2 double‐exp., MYC/BCL2 double expression; NCCN‐IPI, National Comprehensive Cancer Network International Prognostic Index; NGC, nongerminal center B‐cell like.

## 4 Discussion

The need to improve the established prognostic index for predicting the progression of DLBCL remains unmet. Despite being developed over 30 years ago, the IPI stands as a robust tool, initially derived from clinical data of over 1000 patients with diffuse, aggressive lymphomas treated with CHOP or similar chemotherapy regimens [2]. Although the IPI remains effective in the rituximab era, its discriminatory power has waned, particularly among higher‐risk groups [3, 4]. To address this limitation, alternative prognostic scores like the revised IPI and NCCN‐IPI have emerged [4, 5, 7]. However, these adaptations are essentially slight modifications of the original five clinical factors (age, clinical stage, lactate dehydrogenase (LDH) levels, extranodal disease, and performance score) with no additions [4, 5, 6].

In this study, we aim to broaden the prognostic framework by integrating tumor biology into the NCCN‐IPI. We demonstrate substantial improvements by incorporating markers of the tumor microenvironment, specifically stromal FOXC1 and tumor pERK1‐2 expressions, offering a multidimensional approach to DLBCL prognosis. Our results indicate that stromal FOXC1 and tumor pERK1‐2 expression provide stronger prognostic value compared to established factors, including cell of origin and MYC/BCL2 double expression. Incorporating both stromal FOXC1 and tumor pERK1‐2 expressions markedly improved the prognostic accuracy of the NCCN‐IPI, as evidenced by both classical statistics and ML models. These findings are compelling evidence that stromal FOXC1 and tumor pERK1‐2 expression accurately represents the tumor microenvironment of DLBCLs.

From the present study, we noted that high levels of FOXC1‐positive stromal cells and high pERK1‐2‐positive tumor cells within the tumor microenvironment were significantly related to worse prognosis of DLBCL patients. Previous research supports the notion that FOXC1 functions as a negative prognostic factor; elevated expression of FOXC1 has been associated with poor prognosis in multiple malignancies, such as, hepatocellular carcinoma, pancreatic ductal adenocarcinoma, gastric cancer, and breast cancer [51]. Blonska et al., identified *FOXC1* as a target gene of *JUN*, which is linked to the dissemination of DLBCL through interactions with the microenvironment [20]. While they analyzed *FOXC1* expression by DNA microarray on tumor DLBCL cell lines, we analyzed FOXC1 protein expression on DLBCL‐associated dendritic cells, both studies demonstrated that FOXC1 expression was significantly correlated to two or more extranodal sites. FOXC1 was also associated with poor prognostic subtype of basal‐like breast cancers due to its role in mesodermal tissue development [16]. It is also a direct effector of long noncoding RNAs that promote tumor progression and contribute to an immune‐suppressing tumor microenvironment in nonsmall cell lung cancer [15]. These discoveries elucidate the mechanisms by which high FOXC1 expression contributes to worse prognosis in DLBCL. Notably, while previous studies primarily focused on FOXC1 expression in tumor cells, our research emphasizes its expression in stromal cells, where FOXC1 was preferentially expressed compared with tumor cells. Our results indicate potential interactions between tumor cells and microenvironment stromal cells of DLBCL, mediated by FOXC1.

The activation of the ERK1‐2 signaling pathway, often indicated by phosphorylation (pERK1‐2), is crucial in various cellular processes, including proliferation, differentiation, and survival. Dysregulation of this pathway has been implicated in numerous malignancies including DLBCL [21, 22, 23, 24, 25, 26, 27, 28, 52, 53], where aberrant elevated pERK1‐2 signaling contributes to tumor progression and therapeutic resistance. In DLBCL specifically, increased pERK1‐2 expression has been linked to adverse clinical outcomes, such as shorter overall survival and disease‐free survival rates [22, 23]. The therapeutic value of pERK1‐2 targeted treatment has also been explored, with successful destruction of DLBCL cells by inhibition of ERK1‐2 by MAPK inhibitors [28, 29]. These evidences all point to the strong association of the pERK1‐2 pathway in the aggressive behavior of DLBCL.

ML models are relatively new analytical tools that have demonstrated their ability to complement and sometimes surpass the traditional Cox proportional hazards model [31, 32, 33]. ML models offer several notable advantages, such as the ability to capture nonlinear associations, handle complex interactions without explicit programming, and adapt to nonproportional hazards over time. Recognizing these benefits, we employed a multidimensional approach that combines classical statistics with ML models. Specifically, we enhanced the NCCN‐IPI with combinations of stromal FOXC1 and tumor pERK1‐2 as well as cell of origin, MYC/BCL2 double expression, and tumor pERK1‐2. These modifications significantly improved the prognostic accuracy for time to death compared to the original NCCN‐IPI, according to classical statistics. Moreover, these combinations yielded the highest maximum C‐index and the highest average C‐index values among all 16 combinations assessed in ML models. The consistent results across both analytical methods, particularly for the highlighted combinations, reinforce the significance of stromal FOXC1 and tumor pERK1‐2 as crucial prognostic indicators within the DLBCL tumor microenvironment.

In conclusion, the expression of stromal FOXC1 and tumor pERK1/2 was associated with both prognosis and underlying tumor biology, reflecting the tumor microenvironment in DLBCL. Incorporating these markers into the established gold standard prognostic index, NCCN‐IPI improved prognostic accuracy for DLBCL. Although our study is constrained by a relatively small cohort of 92 patients and a limited number of tumor microenvironment markers, the findings underscore the need for larger studies and collaborative efforts employing comprehensive molecular profiling to validate these markers and facilitate their integration into clinical practice. Despite these limitations, the observed improvement in prognostic accuracy with the inclusion of these markers highlights the promising potential of targeting FOXC1‐positive stromal cells and pERK1/2 tumor cells as innovative therapeutic strategies for treating recurrent and aggressive DLBCL.

## Author Contributions

**Ji‐Ye Kim:** conceptualization (lead), data curation (lead), formal analysis (equal), funding acquisition (lead), investigation (equal), methodology (equal), project administration (lead), writing – original draft (equal), writing – review and editing (lead). **Ibadullah Kahttana:** conceptualization (supporting), data curation (supporting), formal analysis (equal), investigation (equal), methodology (equal), writing – original draft (equal), writing – review and editing (equal). **Hyonok Yoon:** conceptualization (supporting), funding acquisition (supporting), investigation (supporting). **Sunhee Chang:** investigation (supporting), writing – review and editing (supporting). **Sun Och Yoon:** conceptualization (supporting), data curation (lead), investigation (supporting), project administration (lead), resources (equal), supervision (lead), writing – review and editing (supporting).

## Ethics Statement

This study was approved by the Institutional Review Board (IRB) of Yonsei University Severance Hospital approved the study (2019‐1925‐001). Written informed consent for participation was waived for this retrospective analysis in accordance with the national legislation and the institutional requirements. The study protocol was conducted according to the Declaration of Helsinki, and local laws and regulations of the Republic of Korea.

## Conflicts of Interest

The authors declare no conflicts of interest.

## Supporting information


Figure S1.

## Data Availability

The data that support the findings of this study are available from the corresponding author upon reasonable request.
