# Supplementary material for: Exploring the Potential of Enhanced Prognostic Performance of NCCN‐IPI in Diffuse Large B‐Cell Lymphoma by Integrating Tumor Microenvironment Markers: Stromal FOXC1 and Tumor pERK1/2 Expression
Source: Cancer Med. 2024 Oct 15;13(19):e70305. doi: 10.1002/cam4.70305 (PMC11475023; doi:10.1002/cam4.70305)

**Supplementary figure 1. C-index heat map of for overall survival predictions by machine-learning**

1.
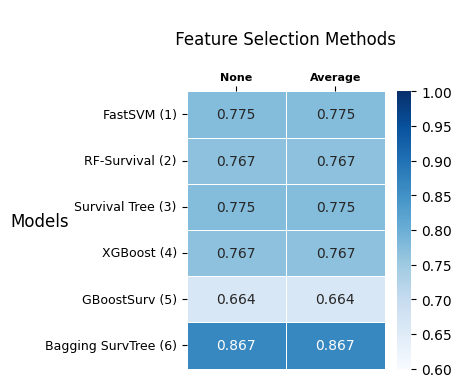
NCCN-IPI
2. NCCN-IPI + stromal FOXC1


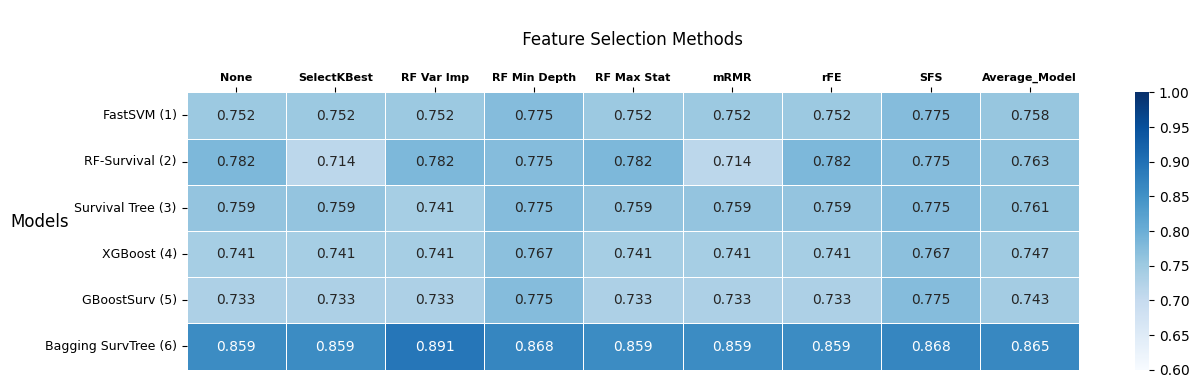


1. NCCN-IPI + tumor pERK1-2


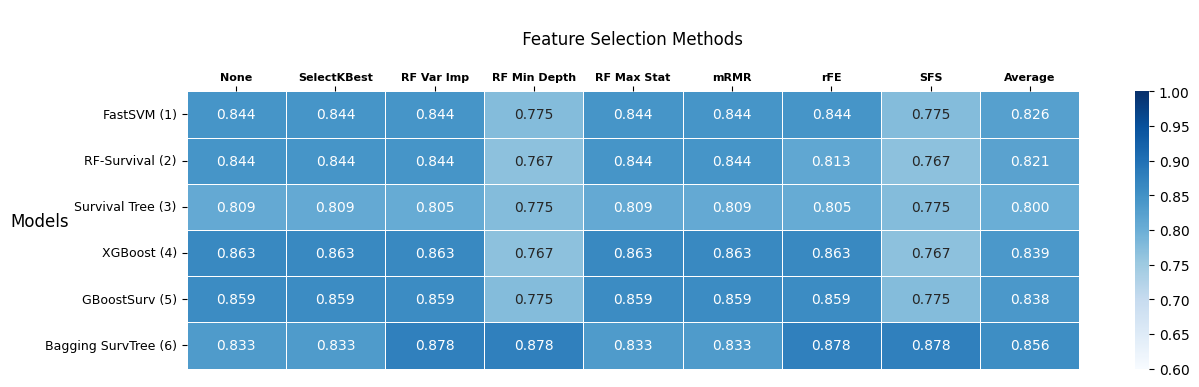


1. NCCN-IPI + cell of origin


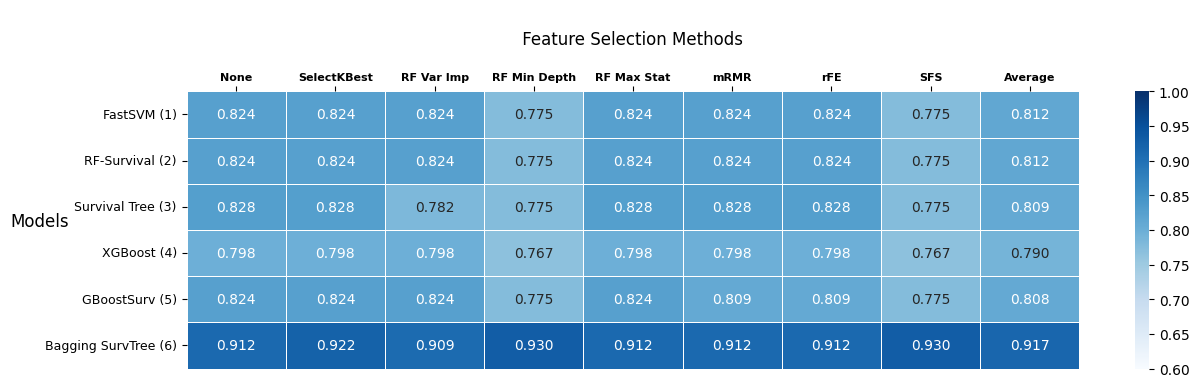


1. NCCN-IPI + MYC/BCL2 double-exp.


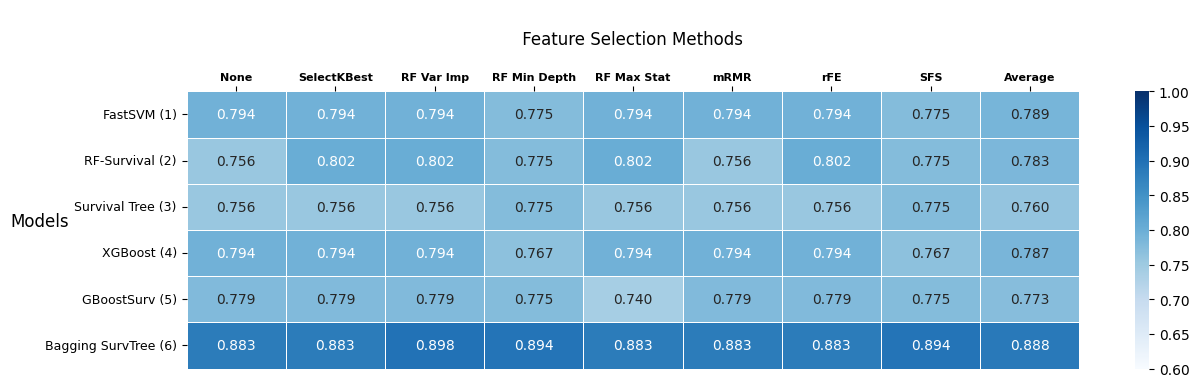


1. NCCN-IPI + stromal FOXC1+ cell of origin


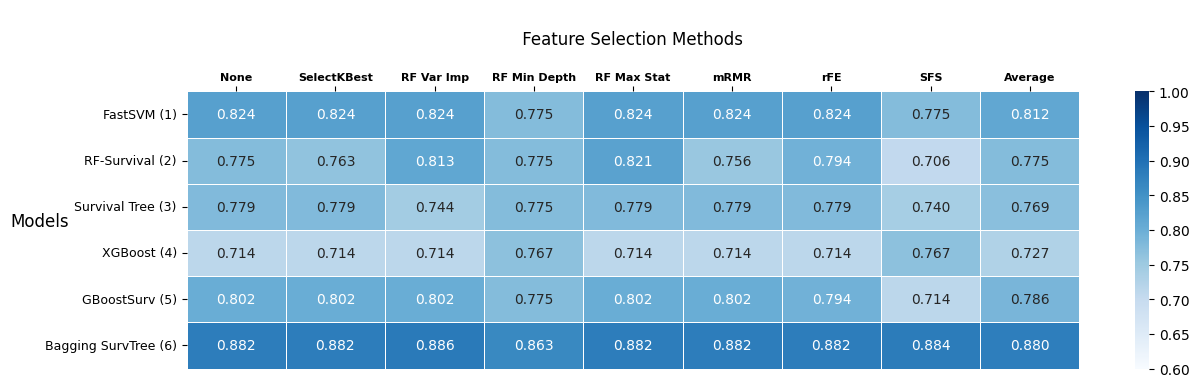


1. NCCN-IPI + stromal FOXC1 + tumor pERK1-2


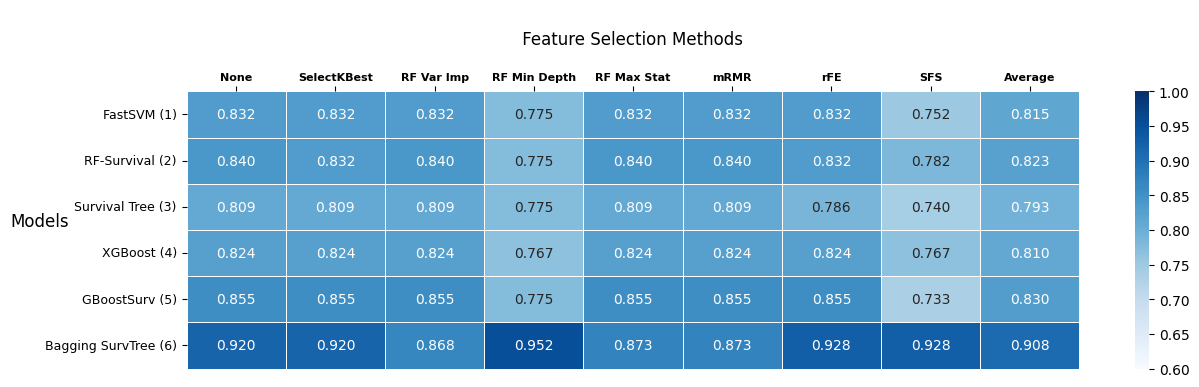


1. NCCN-IPI + tumor pERK1-2 + cell of origin


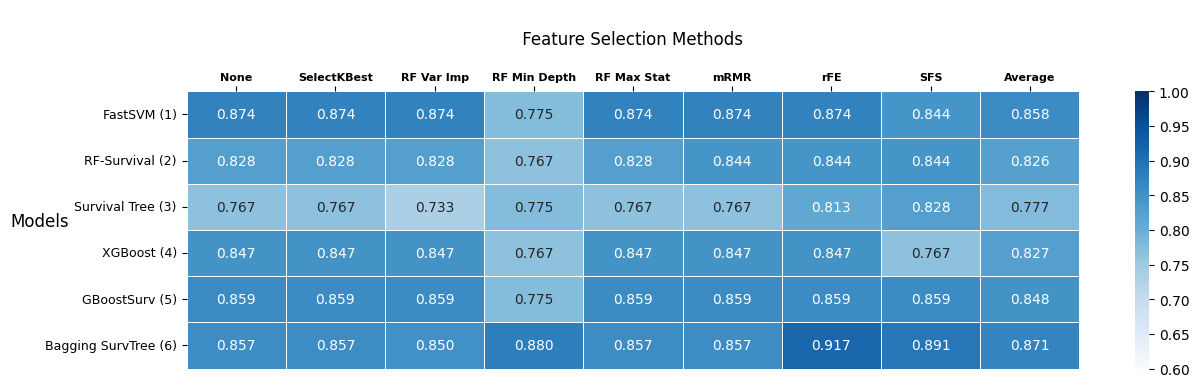


1. NCCN-IPI + stromal FOXC1 + MYC/BCL2 double-exp.


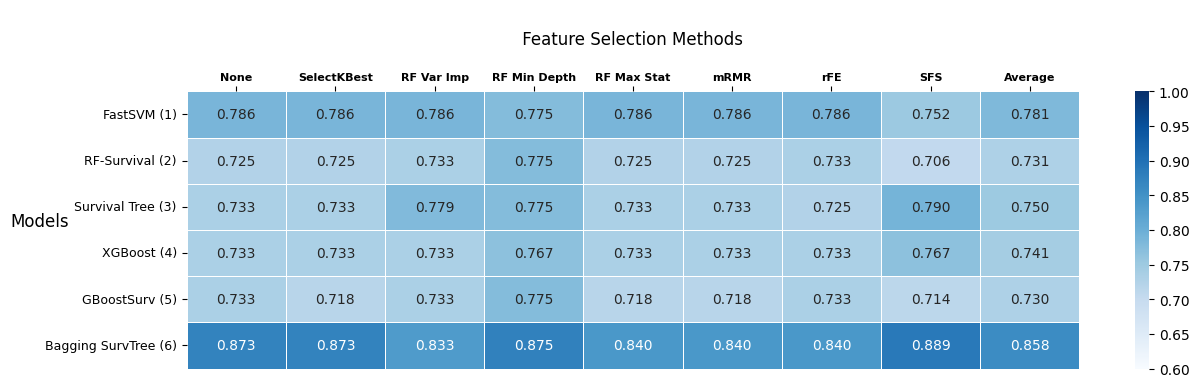


1. NCCN-IPI + tumor pERK1-2 + MYC/BCL2 double-exp.


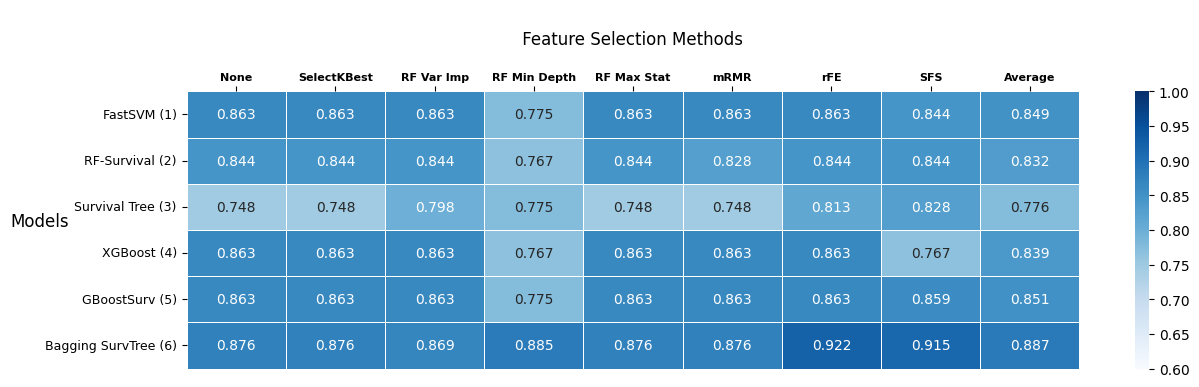


1. NCCN-IPI + cell of origin + MYC/BCL2 double-exp.


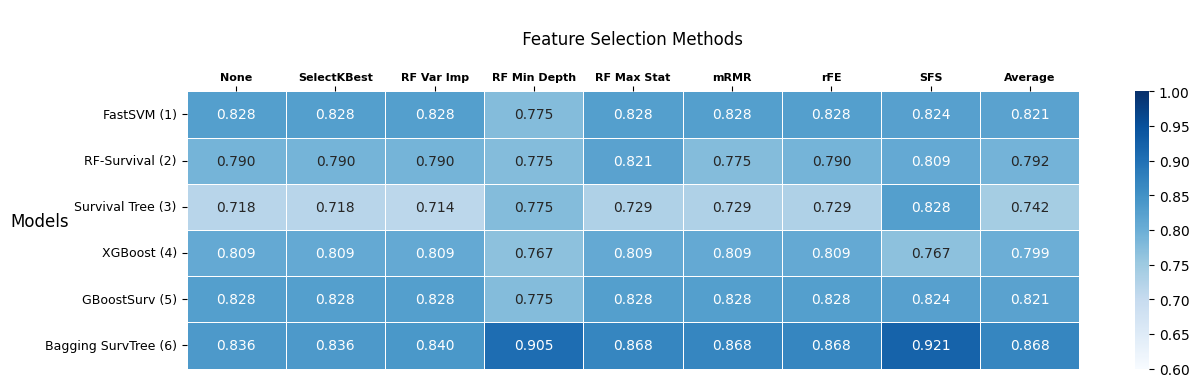


1. NCCN-IPI + stromal FOXC1 + tumor pERK1-2 + cell of origin


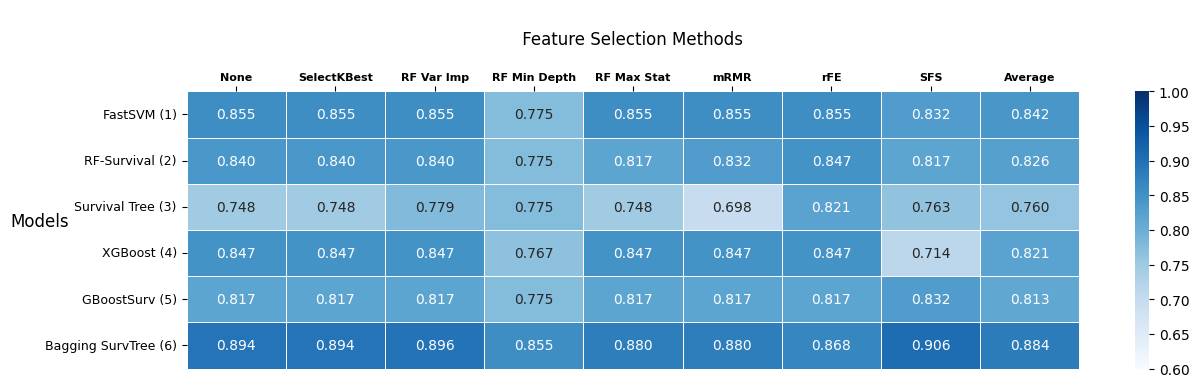


1. NCCN-IPI + stromal FOXC1 + tumor pERK1-2 + MYC/BCL2 double-exp.


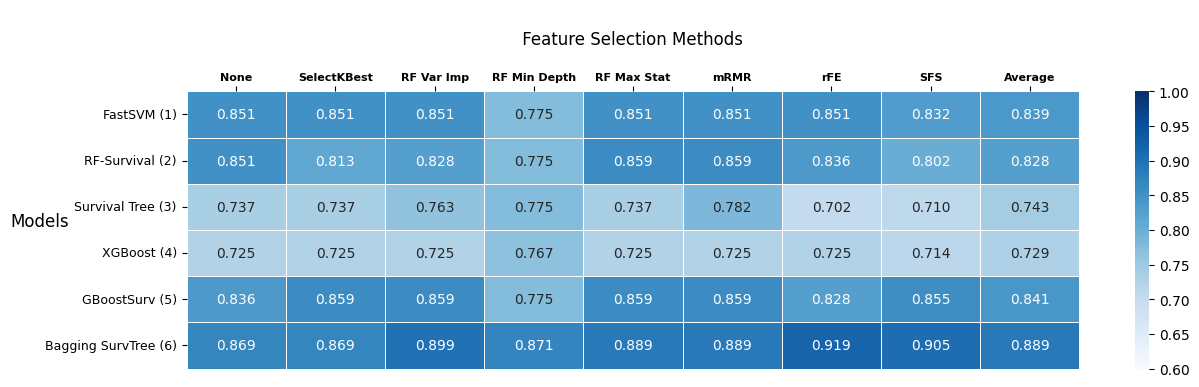


1. NCCN-IPI + stromal FOXC1 + cell of origin + MYC/BCL2 double-exp.


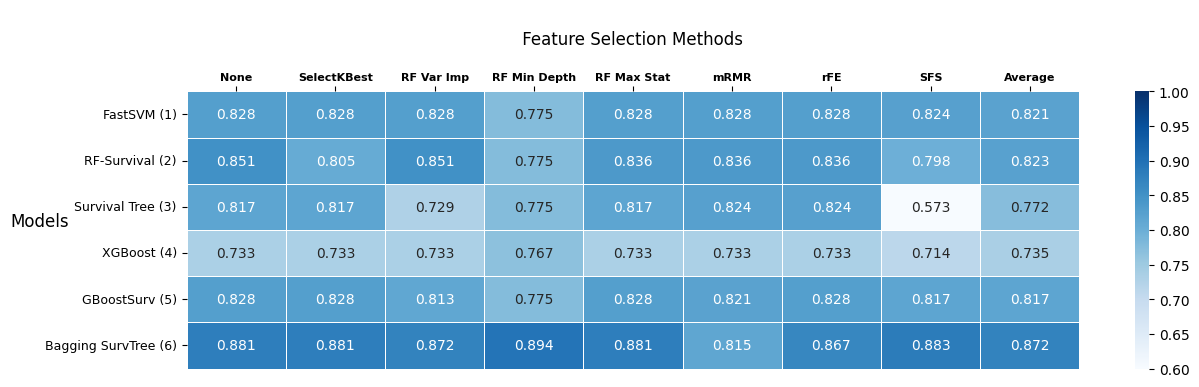


1. NCCN-IPI + tumor pERK1-2 + cell of origin + MYC/BCL2 double-exp.


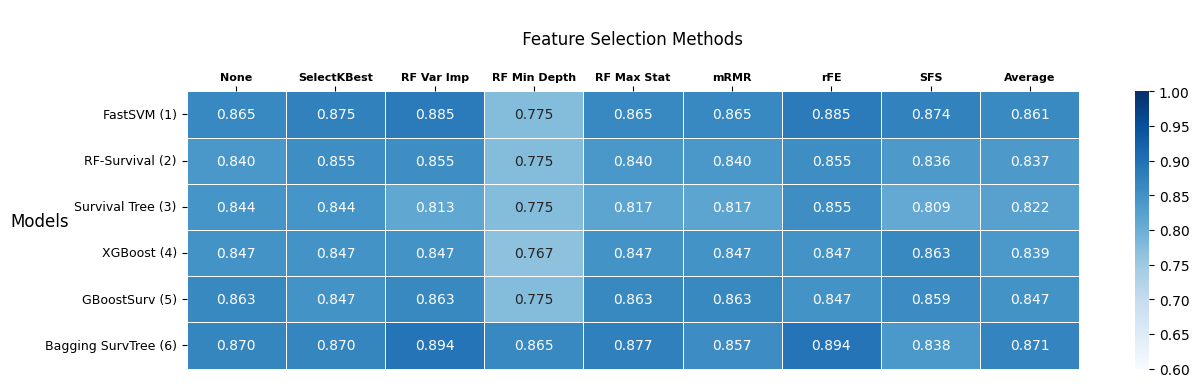


1. NCCN-IPI + stromal FOXC1 + tumor pERK1-2 + cell of origin + MYC/BCL2 double-exp.


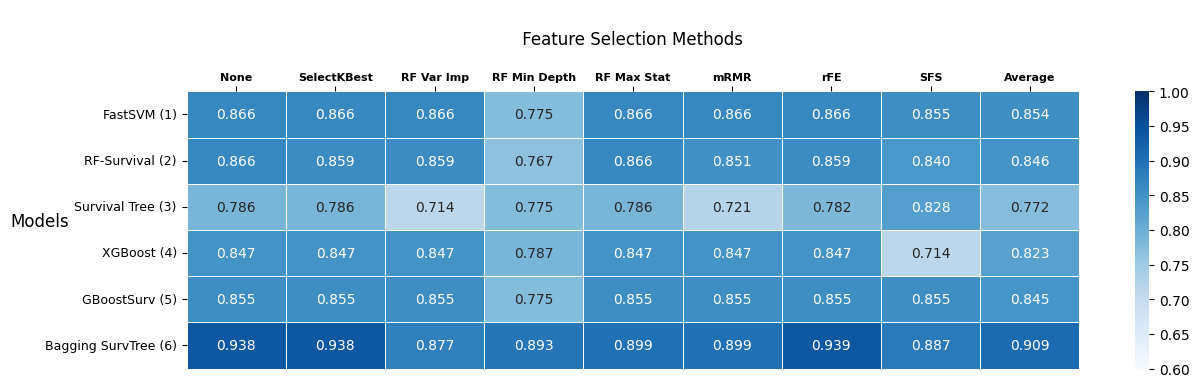

Supplement: Supplementary file 1 — Figure S1. [file CAM4-13-e70305-s001.docx]
